# Emotional and social competencies and perceptions of the interpersonal environment of an organization as related to the engagement of IT professionals

**DOI:** 10.3389/fpsyg.2015.00623

**Published:** 2015-06-10

**Authors:** Linda M. Pittenger

**Affiliations:** College of Business, Embry–Riddle Aeronautical UniversityDaytona Beach, FL, USA

**Keywords:** engagement, emotional and social competencies, interpersonal environment, shared vision, role breadth self-efficacy

## Abstract

There is a dearth of research focused on the engagement of information technology (IT) professionals. This study analyzed the relationship between emotional and social competencies and the quality of the IT professional’s perceptions of the interpersonal environment in an organization as they relate to employee engagement. Validated instruments were used and data was collected from 795 IT professionals in North America to quantitatively analyze the relationship between emotional and social competencies, role breadth self-efficacy (RBSE), with the quality of the IT professional’s perceptions of the interpersonal environment, and those perceptions with employee engagement. The study results indicate that specific emotional and social competencies and RBSE relate differently to the quality of the perceptions of the interpersonal environment. The study also reveals how the quality of the IT professional’s perceptions of the interpersonal environment relates to how much they engage in the organization. The findings indicate that the relationship between achievement orientation and the perceived interpersonal environment was positive and the relationship between influencing others and the perceived interpersonal environment was negative. Understanding such relationships offers much needed insight to practitioners and can benefit organizations that wish to increase the engagement of their IT professionals. The findings also can support practitioners to more effectively select and develop talent with the desired motives and traits. By doing so, organizations can experience increased employee satisfaction, engagement, and retention, resulting in higher productivity, quality, and profitability.

## Introduction

While it is widely agreed that employee engagement is critical to effectively implement strategic goals (Becker and Huselid, 2006), empirical research consistently claims that today’s workers are far from fully engaged and the ‘engagement gap’ is estimated to cost U.S. businesses $300 billion annually in lost productivity (Kowalski, 2003; Bates, 2004; Johnson, 2004). Towers Watson (2012) found that two-fifths of today’s workers are detached, and a quarter completely disengaged, resulting in significant risk to an organization’s productivity and performance goals. Information technology (IT) professionals fare the worst, with only 26% reporting full engagement and 22% admitting to outright disengagement (Treadwell and Alexander, 2011). IT’s performance is plagued by missed deadlines, overrun budgets, and unrealized investments (Ellis, 2009; Johnson, 2009). This begs the question if the high level of disengagement of IT workers is contributing to the numerous performance deficits in the industry and if so, how can IT worker engagement be increased?

The nature of IT work has changed significantly over the past two decades as organizations moved toward flatter, team-based and relational organizing models. This shift in work demands that IT professionals develop more than technical skills. The literature on IT performance has largely ignored the effects of investing in the soft skills (interpersonal skills and teamwork) that IT people need to effectively operate in this new environment (Hitt and Brynjolfsson, 1994; Brynjolfsson and Hitt, 2000). Without these softer skills, it is little wonder why IT professionals are not fully engaged and appear to be working below their potential. Employee engagement is defined as a positive, work-related state of mind exhibited by high levels of energy, dedication, persistence, and happy absorption (Schaufeli et al., 2002). In line with Schaufeli et al.’s (2002) *theory of engagement*, engagement is not conceptualized as a momentary and specific state, rather “a … persistent and pervasive affective-cognitive state not focused on any particular object, event, individual, or behavior.” This research was designed to understand which emotional and social competencies and organizational factors relate to the engagement of IT professionals. The research model was tested on a sample of 795 North American IT professionals using structural equation modeling. The paper begins with a review of the pertinent theoretical foundations and an explanation of the constructs used to articulate a research model and related hypotheses on employee engagement. This is followed by an examination of the research methods deployed, and finally, an analysis and discussion of the findings, limitations, and implications for future research and practice.

## Theory and Hypotheses

### Employee Engagement as Absorption, Dedication, and Vigor

In line with the conceptual definition presented earlier, Schaufeli et al.’s (2002) sub- constructs of engagement (absorption, dedication, and vigor) were used as the mediating variables in the research model. Absorption is defined as a state in which a person is “fully concentrated and deeply engrossed” (Schaufeli et al., 2002, p. 75) in their work. Dedication captures a “sense of significance, enthusiasm, inspiration, pride, and challenge” (Schaufeli et al., 2002, p. 74) and both an emotional and cognitive involvement in a person’s work. Finally, vigor is defined as “high levels of energy and mental resilience while working, the willingness to invest effort in one’s work, and persistence even in the face of difficulties” (Schaufeli et al., 2002, p. 74).

### Interpersonal Environment as an Antecedent to Engagement

The interpersonal environment is considered to be a subset of the organizational environment – defined as the employee’s perception of the practices, policies, and processes of an organization (Ostroff et al., 2003). Research has found both direct (Corporate Leadership Council, 2004) and indirect (Iacono and Weisband, 1997; Jarvenpaa et al., 1998) relationships between the organizational environment and employee engagement, and closely related, employee commitment (Eisenberger et al., 1986). However, less research has focused specifically on the importance of the interpersonal environment. This paper claims that there is a relationship between the IT professional’s perception of the interpersonal elements of the organizational environment and employee engagement.

Boyatzis (2013) claims that the interpersonal environment in an organization is comprised of three dimensions: shared vision, compassion, and overall positive mood. These three dimensions were used in the research model. Shared vision is defined as the degree to which the people in a relationship perceive that they have a shared vision, or desired image of the future. It is proposed that shared vision will positively relate to engagement because when employees have clear direction and confidence in themselves to achieve that vision they are more likely to be engaged in their work. Specifically, the shared nature of a vision will elicit feelings that support the three sub constructs of engagement: excitement and enthusiasm for their work (dedication), a sense of ownership and investment in their work (vigor), and increased absorption in their daily activities.

Compassion is concerned with the degree to which the people in a relationship perceive that they care for and trust each other. Seppala et al. (2013) claim that compassion boosts coworkers’ commitment to the workplace, and level of engagement with their job, resulting in higher productivity levels. While Haidt (2012) states that to be fully engaged in your work and those you are working with, interpersonal distractions such as a lack of trust and care must be alleviated. Thus, when employees are emotionally invested in the success of each other as well as the organization, they will be more engaged. Boyatzis et al. (2013) support this arguing that compassion toward others has a psychological and physiological benefit for both the giver and the receiver. Overall positive mood refers to the degree to which the people in a relationship perceive that they shared a positive view of the present and future. The benefits of such positivity are now well established in social science literature. In particular, positive emotions widen people’s outlook and scope of attention, altering people’s mindsets (Fredrickson and Losada, 2005). In sum, it is hypothesized that:

*Hypothesis 1: There is a positive relationship between the IT professional’s perception of shared vision, compassion and overall positive mood, three sub-constructs of the interpersonal environment and absorption, dedication and vigor, three sub-constructs of employee engagement*.

### Influence of Emotional and Social Competencies on the Interpersonal Environment

Understanding that the organizational environment influences employee engagement is of little use if we do not know how to create such an environment. As discussed earlier, the changing nature of IT work demands that IT workers develop both technical (hard) skills and interpersonal (soft) skills in order to perform at their full potential. Prior research has not identified what specific soft skills are important in the technology organization environment. More specifically, to create an interpersonal environment characterized by shared vision, compassion, and overall positive mood that increases engagement, it is the soft skills rather than the technical skills that will be critical in doing so (Saks, 2006). Thus, in this section, it is hypothesized that a number of specific emotional and social competencies will influence the IT professional’s perception of the organizational environment, and consequently employee engagement.

Boyatzis (1982) defines emotional and social competencies as knowledge, motives, traits, attributes, and skills that are causally related to effective or superior performance in any given job or role. According to Boyatzis’ model, highly effective workers must not only possess intellectual intelligence, but also emotional intelligence. Boyatzis (2001) and Boyatzis et al. (2001/2007) have developed a comprehensive inventory of emotional competencies (self-awareness and self-management), and social competencies (social awareness and relationship management) that together can be used to measure the higher order construct of emotional intelligence. The majority of studies that have used this measure report findings as composites (see for example Boyatzis, 2009; Boyatzis et al., 2012; Mahon et al., 2014; Quinn, 2014), While this approach is useful for understanding overarching relationships between emotional intelligence of various outcome variables, it limits our understanding of which specific competencies are most important, and rests on the assumption that all competencies are required in all environments. To examine this assumption, this study on IT professionals was designed to collect a sufficiently large sample to allow for examination of the relative impact of separate competencies. The competencies included in Boyatzis’ emotional and social intelligence model are summarized in **Table 1**.

**Table 1:** Emotional and social intelligence competencies.

Competency cluster	Competency	Definition
Self awareness	Emotional self awareness (ESA)	Recognizing how our emotions affect our performance and using this is a guide to behavior.
Self management	Achievement orientation	Striving to meet or exceed a standard of excellence.
	Adaptability	Flexibility in handling change.
	Emotional self control	Keeping disruptive emotions and impulses in check.
	Positive outlook	Persistence in pursuing goals despite obstacles and setbacks.
Social awareness	Empathy	Sensing others’ feelings and perspectives and taking an active interest in their concerns.
	Organizational awareness	Reading a group’s emotional currents and power relationships.
Relationship management	Conflict management	Negotiating and resolving conflict.
	Coach and mentor	Taking an active interest in others’ development needs and bolstering their abilities.
	Influencing others	Having a positive impact on others: persuading or convincing others.
	Inspirational leadership	Inspiring and guiding individuals and groups.
	Teamwork	Working with others toward a shared goal.

*Source: adapted from Goleman et al. (2013)*.

In the following section, the specific emotional and social competencies that are believed to influence the IT professional’s perception of shared vision, compassion, and overall positive mood respectively of IT professionals will be examined.

### Emotional Intelligence Antecedents of Shared Vision of IT Professionals

#### Adaptability

Today, organizations are dynamic, complex, and always changing. New leaders come and go and shared visions for the firm change with the new leaders. Such change leads to turbulence, which makes it more difficult to create and maintain a shared vision. Similar to this is the IT organization, which is arguably the fastest changing due to its ever-emerging technologies. IT professionals are both the initiators and recipients of these changes, thus their everyday work requires high levels of adaptability and cognitive flexibility. IT professionals who are high in adaptability are better able to cope with change and turbulence. Therefore, it is expected that IT professionals with high adaptability will perceive a positive relationship to shared vision.

#### Empathy

Information technology professionals are often called nerds, geeks, and other less than complimentary labels. Most are introverts, who are sensitive about their lack of fitting in with others. As a result, they have empathy for others and seek to work with and for those who sense their feelings and perspectives. Pavlovich and Krahnke (2012) claim that empathy enhances connectedness, which occurs through altruistic actions, which promotes pleasurable feelings and harmony and a more expansive, united, state of mind (Pavlovich and Krahnke, 2012). Thus, it is expected that empathy will be perceived to be positively related to shared vision.

#### Organizational Awareness

The IT organization exists to support the functioning of the enterprise and strategic business objectives. The IT professional serves the business and is deeply ingrained in its emotional currents and power relationships. Organizational awareness enables an individual to assess who is able to influence whom, allowing them to appeal to the appropriate person if influence is necessary. IT professionals interact daily with those of influence and in power to understand how technology can provide value. Thus, it is assumed that organizational awareness will be perceived to be positively related to shared vision.

#### Emotional Self-Awareness

Emotional self-awareness refers to a person’s ability to recognize and understand his or her own emotional responses. The ability to understand and connect with others first requires an understanding of self and the ability to regulate emotion. Emotion regulation refers to “the processes by which individuals influence which emotions they have, when they have them, and how they experience and express these emotions” (Gross, 1998, p. 275). The creation of a shared vision requires an ongoing negotiation between self and others. It is through this negotiation over time that shared understanding and ultimately, a shared vision emerges. In order to be emotionally present in these negotiations, individuals must understand and have control over their own emotions. Without such an understanding, a person cannot fully engage and respond to those around them. Thus, it is expected that emotional self-awareness will be positively related to shared vision.

#### Achievement Orientation

An unconscious desire to do better was studied by David McClelland and his colleagues for decades and is called the need to achieve (McClelland, 1961). Although achievement orientation is related to excellence in management and leadership (Boyatzis, 1982; Spencer and Spencer, 1993; Goleman, 1998), it has also been associated with individualistic careers where daily feedback on one’s own performance is clear, like in sales, engineering and IT (McClelland, 1961; Spencer and Spencer, 1993). Such a disposition focused on individual achievements and activities that bring individualized feedback is likely to detract from focusing on people, relationships and shared emotions. Meanwhile, an IT organization operates on the foundation of teams and the individual outputs of IT professionals are typically integrated with outputs of their peers and others in the business. Thus, to be most effective, the individual’s tasks in IT should support common or shared goals, and by extension a shared vision. At the same time, working with others in the context of a shared vision should help provide a meaningful context for the individualistic activity of the IT professional. This shared vision helps the IT professional understand how their individual work contributes to the firm’s success. Given this, it is expected that achievement orientation will be linked to effectiveness through shared vision.

#### Conflict Management

Information technology professionals lack interpersonal skills and creating a shared vision takes patience, strong interpersonal behaviors, negotiation, and even conflict management skills. It is expected that the ability to negotiate differences to arrive at unified solutions is particularly pertinent in the early stages of vision formation. Conflict management requires open communication, which is atypical of the introverts that populate IT. It is anticipated that conflict management to be perceived by IT professionals to be negatively related to shared vision.

#### Influencing Others

Information technology organizations are dominated by introverted professionals who have difficultly communicating and take action based on what they think rather without considering how others feel (Capretz, 2002). Efforts to communicate and attempts to influence others may backfire in reality and detract from the support of a shared vision, rather than the act of promoting it. Thus, it is expected that influencing others will be perceived to be negatively related to shared vision.

In sum, the following relationships between emotional and social competencies and shared vision are hypothesized:

*H2: Individual emotional and social competencies of adaptability, empathy, organizational awareness, emotional self-awareness, and achievement orientation will positively and conflict management and influencing others will negatively affect the IT professional’s perception of shared vision*.

### Emotional Intelligence Antecedents of Compassion of IT Professionals

Compassion in organizations makes people feel seen and known; helps them feel less alone, and positively affects emotional connections between people at work (Frost et al., 2000). Compassion exists when individuals of a system collectively notice, feel, and respond to pain experienced by others (Kanov et al., 2004). Since compassion is associated with a range of positive attitudes, behaviors, and feelings in organizations (Dutton et al., 2002; Lilius et al., 2003), investigating how emotional and social competencies might affect compassion is important and should be examined (Boyatzis and Goleman, 1996; Boyatzis, 2001; Boyatzis et al., 2001/2007).

#### Adaptability

Compassion requires people to notice, empathize, and act on the needs of others (Boyatzis et al., 2013). This requires cognitive flexibility in order to recognize that situations in which one person may be in need are not necessarily those in which another may be in need. This recognition is the first step in compassion – without it, one cannot be compassionate. Upon noticing another in need, a person needs to act on that need. Due to the cognitive flexibility of adaptable individuals, they are able to consider wide range of potential actions, which increases the likelihood that they are able to respond appropriately. Adaptable individuals are also more capable of changing their own behavior or circumstances to accommodate the needs of others (Aronoff et al., 1994). Thus, it is anticipated that there is a positive relationship between adaptability and compassion.

#### Empathy

Empathy is considered as a distinct, yet closely connected construct to compassion (Kanov et al., 2004). Compassion is considered to be a broader construct than empathy, requiring a deeper connection to the person in need (Dietze and Orb, 2000). While empathy primarily involves noticing and feeling the need of another, compassion takes this a step further by acting on this need (Kanov et al., 2004; Boyatzis et al., 2013). Prior research has linked empathy to increase helping behavior (Betancourt, 1990). Since IT professionals are seen as sensitive and caring introverts, it is anticipated that IT professionals will perceive that empathy and compassion are positively related.

#### Organizational Awareness

Compassion is commonly conceptualized as a three-part process consisting of noticing, feeling and responding to another’s needs (Kanov et al., 2004). While organizational awareness may increase the ability of a person to notice the need of another by paying attention to the emotional currents in the organization, the key role in organizational awareness in increasing perceived compassion in organizations is in the ability to respond. To be successful, IT professionals must understand the formal and informal power distributions and networks in and between organizations and how to use this to gain access to resources to respond to the needs of the business. Therefore, it is expected that organizational awareness will be perceived to have a positive relationship with compassion.

#### Emotional Self-Awareness

In order to detect the need or suffering of another person, we are often forced to rely on our ability to read a person’s emotional state. Further, in making sense of the observed emotions or behavior of a colleague, we rely on our own experience (Weick, 1995) to understand emotional responses and reactions to assist us to notice and respond to the needs of others. Since IT professionals are introverted and very much in touch with their emotions, it is anticipated that emotional self-awareness will be perceived to have a positive relationship with compassion.

#### Achievement Orientation

Personality tests reveal that IT professionals are more achievement oriented than the general population (Woodruff, 1980; Wynekoop and Walz, 1998) and gain recognition through their expertise (Capretz, 2002). The work of IT is often innovative and comes with risk of failure. Since IT professionals identify closely with the work that they do (Pratt et al., 2006), they are more individualistic and therefore less focused on others. Given this, it is expected that achievement orientation will be perceived to negatively relate to shared compassion.

#### Conflict Management

Information technology professionals often find themselves in conflict with those in business units over approaches to development and/or requirements gathering. The ability to manage conflict in a positive way reduces the pain experienced by those involved in the conflict, and thus will likely increase perceptions of shared compassion. Compassion does not presume the absence of conflict; rather, a person with good conflict management skills can use compassion to reduce the intensity of the conflict. Thus, it is hypothesized that conflict management will be perceived to be positively related to compassion.

#### Influencing Others

Influencing others is the use of power to get others to comply with one’s wishes or desires (Boyatzis, 1982, 2009; Spencer and Spencer, 1993). The use of this competency will likely occur when there is a power distance (i.e., boss and subordinate) or an influence task (i.e., selling something to another person). As a result, it is unlikely that more use of Influencing Others competency would be related to closer relationships. When added to the observation that the introverted IT professional is energized by an internal world of ideas, not emotion and not communicating with others (Eve-Cahoon, 2003), it is expected that influencing others will be negatively related to the IT professional’s perception of shared compassion.

In sum, the following relationships between emotional and social competencies and compassion are hypothesized:

*H3: Individual emotional and social competencies of adaptability, empathy, emotional self-awareness, achievement orientation, conflict management, and organizational awareness will positively and influencing others will negatively affect the IT professional’s perception of compassion*.

### Emotional Intelligence Antecedents of Shared Positive Mood of IT Professionals

There is a plethora of literature claiming that one’s mood at work affects behavior. For example, George (1991) found that a positive mood at work fosters pro-social organizational behaviors. In the next section, existing theory will be discussed, examining how emotional and social competencies influence the perception of overall positive mood.

#### Adaptability

The ability to adapt both to bad, good, and challenging circumstances is expected to positively relate to overall positive mood. Particularly in the fast changing technical environment, IT professionals who are resistant to change are unlikely to experience organizational life in a positive way. Thus, it is anticipated that for IT professionals, adaptability will be perceived as having a positive relationship with overall positive mood.

#### Empathy

Empathy involves sensing others’ feelings and perspectives and taking an active interest in their concerns. Empathy has been found to lead people to attribute negative behavior to situational factors rather than dispositional factors (Regan and Totten, 1975) and increase helping behavior (Betancourt, 1990). The attribution of negative behavior to situational, rather than personal factors decreases the intensity of the negative response for the person who is displaying empathy, thus improving their mood. For the receiver of the empathy, knowing that someone in the organization understands how you feel and is willing to see your perspective would also result in higher levels of positive mood. IT professionals work within a strong social identity group, which promotes respect and understanding, amongst peers. Therefore, it is expected that empathy will be perceived to have a positive relationship with overall positive mood.

#### Organizational Awareness

A positive relationship exists between organizational awareness and an employees’ commitment for organization success (Gagnon et al., 2014). Organizational awareness is about understanding the power and influence in the group. Membership in IT is important to IT professionals, mimicking that of a fraternity, seeking inclusion and acceptance to belonging to the ‘in-group’. Belonging is a basic psychological need (Baumeister and Leary, 1995) and is a basic, formidable, and extremely pervasive motivation. Therefore organizational awareness can lead to the fulfillment of our need to belong and is expected that IT professionals would perceive it to be overall positively related to positive mood.

#### Emotional Self-Awareness

The technology industry is dynamic and complex. With a constant flow of emerging trends and rapidly changing technologies, IT professionals are forced to constantly examine and upgrade their skills (Ang and Slaughter, 2000; Agarwal and Ferratt, 2002).

Information technology professionals regularly evaluate themselves against their peers. To maintain relevance and sustain marketable skills, IT professionals must reskill and reskill and reskill. One day their skills are in demand and they are being paid bonuses for their skills (Cobol Programmers in 2000) and the next quarter, the skills are no longer needed. The emotional swings of relevancy wreak havoc on the IT professional’s emotions. Thus, it is expected that self-awareness will be perceived as being negatively related to overall positive mood.

#### Achievement Orientation

Information technology professionals take much pride in demonstrating their competence to others. Employees with a performance-approach orientation are motivated to demonstrate superior competence relative to others and to obtain favorable judgments of their achievements (Elliot, 2005). Preenan et al. (2014) found that on high-challenging tasks, employees had a higher positive mood and concluded that a performance-approach orientation elicits a positive-activating mood. Thus, it is expected that the achievement orientation will be perceived as being positively related to overall positive mood.

#### Conflict Management

Conflict management is the process of reducing the negative aspects of conflict while increasing the positive aspects of conflict. By managing conflict, the aim is to enhance learning and boost group outcomes, such as employee effectiveness in an organizational setting (Rahim et al., 1992). Emotions play a role in employee performance, particularly on creative tasks, as those fulfilled by IT professionals. Since the work in IT typically occurs in teams, there is potential conflict and competition for accepted ideas, a part of the creative process. Such conflict management promotes innovation and constructive contention, with likely better outcomes. Thus, it is hypothesized that conflict management will be positively related to overall positive mood.

#### Influencing Others

Information technology professionals are characteristically introverted and most likely to minimize both internal and external stimuli. They maintain a strong identity with individuals that are similar to them and do not actively seek to neither build relationships nor influence others. Kahnweiler (2013) claims that biggest challenge for an introvert is losing confidence and feeling powerless, further asserting that introverts try to influence others by mirroring colleagues, which is tiresome, unsustainable and usually ineffective. Given this, it is expected that influencing others would be perceived as being negatively related to overall positive mood.

In sum, the following relationships between emotional and social competencies and overall positive mood are hypothesized:

*H4: Individual emotional and social competencies of adaptability, empathy, organizational awareness, achievement orientation, and conflict management will positively and influencing others and emotional self-awareness will negatively affect the IT professional’s perception of overall positive mood*.

### Role Breadth Self-Efficacy as an Antecedent of Shared Vision, Compassion, and Overall Positive Mood of IT Professionals

Parker’s (1998) *theory of Role Breadth Self-Efficacy (RBSE)* refers to employees’ perceived capability to carry out a broader and more proactive set of work tasks that extend beyond prescribed technical requirements. Further, Parker claims that those that do engage in behaviors that span beyond their defined roles and assume broader responsibilities essential for organizational success are seen as more valuable (Parker, 1998). The theory of RBSE focuses on emotional behaviors presumed to change in response to the changing conditions of the organization environment (Parker, 1998). This is further supported by Den Hartog and Belschak (2007) who argue that one’s organizational attachment is an important motive for an employee’s engagement. Such findings support the intent of the study by examining how RBSE might have a relationship with the interpersonal environment in an organization (vision, compassion, and overall positive mood).

Information technology professionals place significance on how well their self-image aligns with what they see as representative of the greater group (Ely, 1995; Alvesson et al., 2001) and when a high level of perceived similarity exists, they may be more satisfied with and have a higher positive evaluation of the organization. RBSE should be positively related to shared vision because perceiving that there is a shared vision makes employees more likely to believe that they know how to contribute to organizational goals by going beyond the limits of their roles. The shared vision, in a sense, provides a road map for moving into uncharted territory.

Technical self-efficacy as it relates to the IT professional focuses on the level at which employees believe in their abilities to perform in a given situation (Webster and Martocchio, 1992), while social identity theory (Tajfel, 1978) is defined as the part of an individual’s self-concept derived from membership in a group where value and emotional meaning is attached. Since IT individuals value their skills and expertise, they seek others and choose situations where they can demonstrate their competence (Judge, 2002). RBSE should be positively related to compassion because in a supportive work environment, employees will expect that they will receive help, if necessary, if they go beyond the confines of their role. They also will be less afraid of failing if they do so.

Role breadth self-efficacy relates to an employee’s need to feel confident in their ability to engage in proactive behavior (Parker, 1998). Loundsbury et al. (2012) argue that technically oriented professionals are open to new and radical ideas, very willing to experiment, enjoy variety of tasks, and are inclined to seek out novel experiences. RBSE should be related to overall positive mood because people tend to be more expansive in their thinking and behavior when their mood is positive. Thus, working in a setting where the mood is positive will make employees more likely to go beyond the confines of their roles.

In sum, it is hypothesized that RBSE will positively affect the IT professional’s perception of shared vision, compassion, and overall positive mood:

*H5: RBSE will positively affect the IT professional’s perception of shared vision, compassion and positive mood, the three sub-constructs of the interpersonal environment*.

**FIGURE 1:**
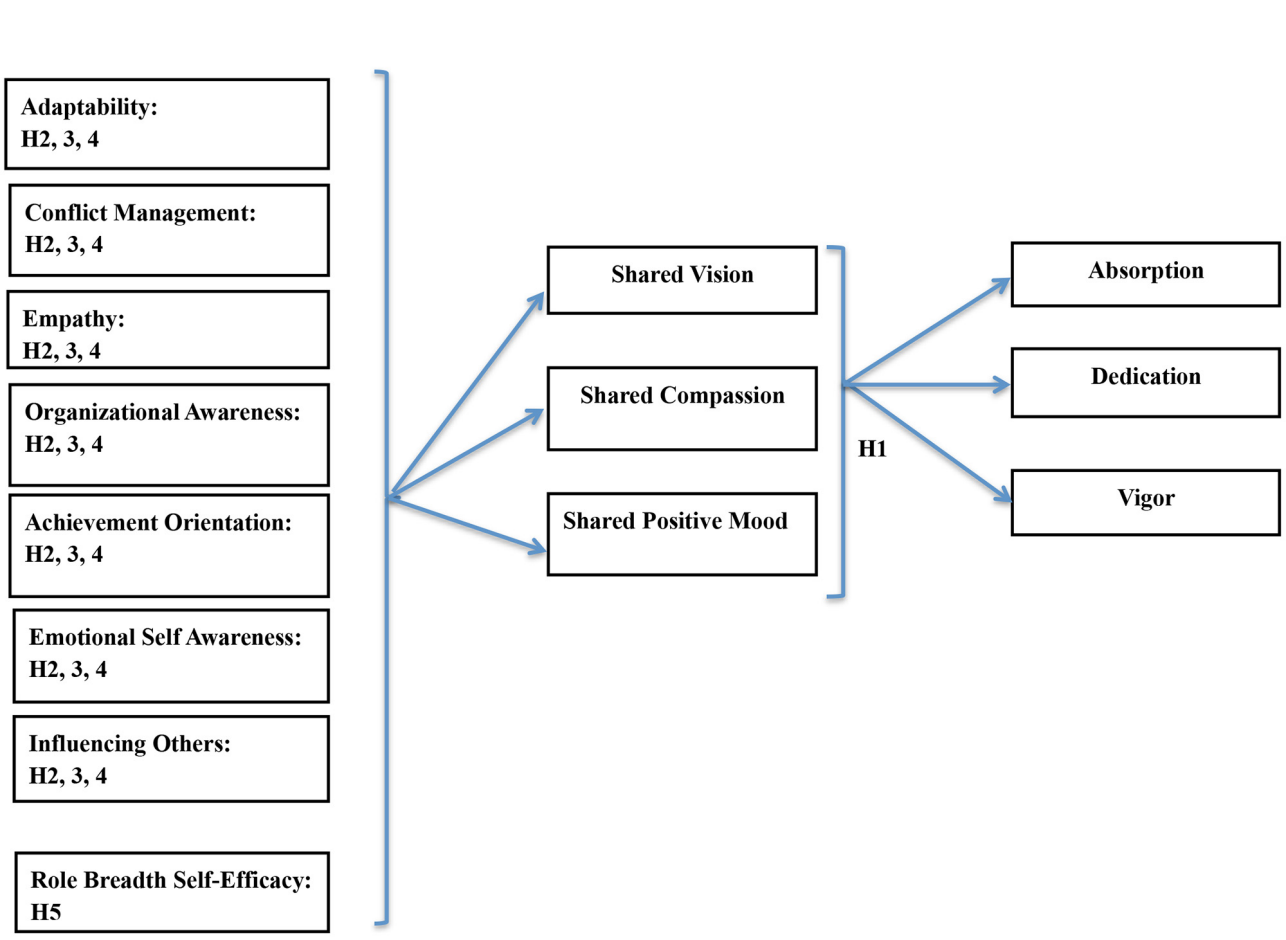
**The mode and hypotheses predicting engagement**.

The research model, as shown in **Figure 1**, visually articulates the flow of this research. Hypothesis 1 represents the IT professional’s perception of the interpersonal environment through the use of three sub-constructs; shared vision, compassion, and overall positive mood and how they may relate to employee engagement, which is represented by three constructs; absorption, dedication, and vigor. Hypotheses 2–4 represent specific emotional and social competencies (adaptability, conflict management, empathy, organization awareness, achievement orientation, emotional self-awareness, and influencing others) and how they may relate to the IT professional’s perception of the interpersonal environment (shared vision, compassion, and overall positive mood). Hypothesis 5 is represented by RBSE and how it may relate to the IT professional’s perception of the interpersonal environment (shared vision, compassion, and overall positive mood).

## Research Method

### Operationalization of Construct

The operationalization of the construct drew mainly from four validated instruments that measured the independent variables (RBSE and emotional and social competencies), interpersonal environment and the dependent variable (engagement).

To measure engagement, the Utrecht Work Engagement Scale (UWES; Schaufeli et al., 2002) was used. Three sub-constructs (dedication, vigor, and absorption) reflect the dimensions of engagement and its 17 items are scored on a 7-point Likert scale ranging from (Never Almost Never, Rarely, Sometimes, Often, Very Often, Always). The reliability and the factorial validity of the UWES (Schaufeli et al., 2002) are suitable. The Cronbach ***a***lpha reliability is as follows: Vigor (0.83), Dedication, (0.90), and Absorption (0.88). Sample questions from the UWES include:

• At work, I feel full of energy.
• I am enthusiastic about my job.
• When I am working, I forget everything else around me.

The perception of the interpersonal environment was measured by the positive and negative emotional attractors (PNEAs) instrument, which Boyatzis (2006, 2013) claims to determine the perspective of the self-organizing process, sometimes adapting to existing conditions and/or to new, emergent conditions. The PNEA construct consists of 20 questions. The construct has three sub-constructs: shared vision, compassion, and overall positive mood. The subscales of the construct are scored on a five point Likert scale (Strongly Agree, Somewhat Agree, Neither, Somewhat Disagree, and Strongly Disagree). The Cronbach alpha reliability is as follows: Vision (0.905), Compassion, (0.853), and Overall Positive Mood (0.909). Lists of questions are summed to provide a score for each sub-construct and then all three sub-constructs are summed to provide a PEANEA score.

Sample questions include:

• I do not feel trusted by my colleagues.
• I feel trusted by my colleagues.
• I care about my colleagues at work.
• I do not care about my colleagues at work.

The Emotional and Social Competency Inventory (ESCI-U; Boyatzis and Goleman, 1996) is a 72-item instrument used to measure the dimensions of emotional and social intelligence. The self-report version of the ESCI-U was used for the study. Seven of the 14 competencies of the ESCI-U were used in the study. Half of the ESCI-U competencies were not used. This was due to a concern regarding the length of the study survey instrument. Competencies were eliminated based on their perceived lack of applicability to employee engagement. This included two cognitive competencies (systems thinking and pattern recognition), two emotional competencies (emotional self-control and positive outlook), and three social competencies (coach and mentor, inspirational leadership, and teamwork). Those included in the study were: adaptability, conflict management, empathy, organization awareness, achievement orientation, emotional self-awareness, and influencing others. The ESCI-U instrument consists of a multi-item scale with a six-point response format (Don’t know, Never, Rarely, Sometimes, Often, and Consistently).

Sample questions include:

• When resolving conflict, deescalate the emotions in the situation.
• Initiate actions to improve own performance.
• Understand the informal structure in the organization.

The questions in the ESCI-U instrument (Boyatzis and Goleman, 1996; Boyatzis et al., 2001/2007) demonstrate the following Cronbach alpha:

Emotional Intelligence Dimension:

– Emotional Self Awareness: recognizing one’s emotions and their effects (six items): 0.771.
– Achievement Orientation: striving to improve or meeting a standard of excellence (six items): 0.705.
– Adaptability: flexibility in handling change (six items): 0.752.

Social Intelligence Dimension:

– Empathy: sensing others’ feeling and perspectives, and taking an active interest in their concerns (six items): 0.725.
– Organizational Awareness: reading a group’s emotional currents and power relationships (six items): 0.764.
– Influencing Others: wielding effective tactics for persuasion (six items): 0.746.
– Conflict Management: negotiating and resolving disagreements (six items): 0.636.

To assess RBSE, Parker’s (1998) instrument was used. The instrument demonstrates a Cronbach alpha of 0.96. The 10-item instrument contains a multi-item scale with a response format (1-not at all confident to 5-very confident) that measures one’s confidence to perform tasks tapping affective elements, such as proactivity and interpersonal and integrative competencies. Scores are aggregated to form a single scale. Sample questions from the construct include:

How confident would you feel:

• Analyzing a long term problem to find a solution.
• Representing your work area in meetings with senior management.
• Writing a proposal to spend money in your work area.

### Sample

The cross-sectional study was administered online and data was collected from May through August 31, 2011. The study was approved by Case Western Reserve University’s IRB. Data was collected through online surveys from IT professionals employed at global companies. Several professional IT associations were contacted for survey participants, who distributed the survey to their members. Participants were contacted via email, inviting them to participate in the study by clicking on a survey link provided. Several procedures were used to ensure high response and completion rates. This included: (a) a cover letter explaining the need for the scholarly research and the critical role of practitioners in to create useful knowledge; (b) assurance of anonymity and individual and organizational confidentiality, and (c) access to completed survey results. Two reminder emails were sent, one email was sent after 2 weeks and a second email after 4 weeks. Since the surveys were distributed through online mechanisms, it was not possible to determine the response rate, non-response bias or to draw conclusions on the reasons for those who dropped out. Qualtrics, a web platform was used to host the survey. A total of 1,143 IT professionals completed the survey. Accounting for returned emails and incomplete responses, 795 usable surveys were included in the study, yielding a 70.0% rate for completed surveys.

### Demographics

**Table 2** informs us of demographics of the study’s respondents. The age of respondents ranged from under 30 to over 60 years of age. Specifically, 13.5% were under 30, 24% are ages 31–40, 29.4% are ages 41–50, 25.7 are ages 51–60, and 6.6% are over the age of 60. In our sample, female participants (29.4%), paled in comparison to males (69.5%). Further, individual contributors represented a little over half (54.2%) and managers (45.3%) of the sample. With respect to experience, 42% had over 20 years of experience and fewer than 2% of IT professionals have less than 1 year. IT professionals with 1–4 years experience represented 10.8%, 12.5% of respondents had 5–10 years, 18.6% had 11–15 years, and 16–20 years of experience consisted of 13.9%.

**Table 2:** Characteristics of respondents (*n* = 795).

	*N*	%
***Age***		
<30 years	107	13.46
31–40 years	191	24.02
41–50 years	234	29.44
51–60 years	204	25.66
Over 60 years	52	6.60
No response	7	<1.0
***Gender***		
Female	235	29.40
Male	555	69.50
No response	5	<1.0
***Job role***		
Individual contributor	431	54.21
Manager	360	45.28
No response	4	<1.0
***Experience***		
Less than 1 year	15	1.88
1–4 years	86	10.81
5–10 years	99	12.45
11–15 years	148	18.60
16–20 years	11	13.88
Over 20 years	436	42.38

## Measurement Model

### Data Screening

Relevant statistical assumptions necessary for subsequent analyses were checked and no violations of assumptions were uncovered. The data screening included handling missing data and addressing outliers and influentials. The analyses showed that the items comprising the RBSE, ESCI-U, PNEA, and UWES were normally distributed around their mean. After reviewing all of the data, a couple of outliers were found that were then removed because of cross loadings and low primary loadings.

### Exploratory Factor Analysis and Confirmatory Factor Analysis

Exploratory factor analysis (EFA) was utilized to see how many factors would explain the patterns among the interrelationships of the items and reduce the number of variables into more manageable factors and examine the convergent and discriminant validity of the constructs. First, 98% of items correlated at least 0.30 or higher with at least one other item, suggesting reasonable factorability. Second, the Kaiser-Meyer-Olkin measure of sampling adequacy was 0.952, above the recommended value of 0.60, and Bartlett’s test of sphericity was significant (32476.489, *p* < 0.001). There were 11 non-redundant residuals with absolute values greater than 0.05. The diagonals of the anti-image correlation matrix were all over 0.50, supporting the inclusion of each item in the factor analysis. Finally, the communalities were all above 0.40 further confirming that each item shared some common variance with other items. There were a significant number of correlations greater than 0.30 were observed, suggesting non-orthogonality. The analysis was continued with an oblique rotation using principle axis factoring (PAF). A promax rotation provided the best-defined factor structure. A factor-loading threshold of 0.40 was set (Hair et al., 2010) and the results showed all items had primary loadings over 0.60 with low and cross loadings of 0.30 or above. Due to both low and cross loading, the variables of emotional self-awareness were sequentially deleted from the analysis until an acceptable model emerged^1^. Other solutions were examined, however, the 14 factor solution, which explained 63.895% of the variance, was preferred because of its theoretical support, the ‘leveling off’ of Eigen values on the scree plot after 14 factors, and the number of primary loadings on their hypothesized factors. A confirmatory factor analysis (CFA) was conducted in AMOS. Using the dataset, significance and several model fit measures were tested. The original measurement model had 100 variables associated with 15 constructs. The Browne–Cudeck criterion (BCC) test of close fit was used and the BCC value was compared across the hypothesized model (Browne and Cudeck, 1993). The 90% confidence level was 0.035–0.037, lower than the saturated model, suggesting a good fit (Floyd and Widaman, 1995). Steiger and Lind’s (1980) root mean square error of approximation (RMSEA) with a 90% confidence interval was used to reflect both the fit and parsimony of the model at hand. The RMSEA was 0.035 and had a PCLOSE of 1.000. The non-normed fit index (NNFI; Tucker and Lewis, 1973), the comparative fit index (CFI; Bentler, 1990), and incremental fit index (IFI) as other goodness-of-fit measures that reflect the proportionate improvement in fit of the measurement model over a more restricted baseline model were used. The NNFI, CFI, and IFI all were “close to 0.96” indicating satisfactory fit (Hu and Bentler, 1998).

### Validity and Reliability

Tests were conducted to evaluate the convergent and discriminant validity and the reliability of reflective measures. Those loadings that exceeded 0.70 on their respective factors were construed as indicative of convergent validity (Straub et al., 2004). Average variance extracted (AVE) ranged from 0.51 to 0.77, exceeding the recommended threshold of 0.50 (Fornell and Larcker, 1981) indicating acceptable convergent validity for each construct. Composite scale reliability ranged from 0.66 to 0.95, exceeding the recommended cutoff value of 0.70 (Nunnally, 1978). The correlation matrix indicated that the requirements of discriminant validity were satisfactorily achieved. Only three discrepant correlations out of a possible 98 were indicated. A second test of discriminant validity of individual items is implicit if they load higher on their own respective construct than on any other latent variable (Gefen et al., 2000; Straub et al., 2004). This requirement was met, with only two discrepancies among a possible 69. The internal consistency of the measures established the reliability by examining the Cronbach alpha and composite reliability, for each construct. Only minor issues are apparent in a few of the variables: adaptability, conflict management, empathy, and organization awareness are just below the 0.7 threshold for reliability, but this is justifiable as there are only two items for each of these factors. **Table 3** and **Table 4** indicate the validity and reliability and correlation results.

**Table 3:** Validity and reliability (*n* = 795).

	Reliability			Variance		
Construct	Items	Cronbach alpha	Composite	Average extracted	Maximum shared	Average shared
***Organizational environment***						
Vision	7	0.91	0.90	0.58	0.74	0.15
Compassion	3	0.82	0.83	0.63	0.32	0.09
Overall positive mood	6	0.94	0.94	0.71	0.74	0.15
***Emotional/social competencies***						
Adaptability	2	0.69	0.69	0.53	0.38	0.16
Achievement orientation	4	0.81	0.82	0.53	0.38	0.22
Conflict management	2	0.70	0.70	0.54	0.37	0.14
Empathy	2	0.67	0.66	0.49	0.34	0.16
Emotional self awareness	4	0.81	0.81	0.51	0.37	0.15
Influence	2	0.73	0.73	0.58	0.19	0.07
Organization awareness	2	0.64	0.68	0.52	0.25	0.11
***Role breadth self efficacy***						
RBSE	7	0.89	0.91	0.55	0.27	0.14
***Engagement***						
Absorption	6	0.95	0.95	0.77	0.76	0.19
Dedication	6	0.94	0.94	0.72	0.51	0.24
Vigor	4	0.87	0.86	0.61	0.76	0.21

**Table 4:** Correlation matrix (*n* = 795).

	1	2	3	4	5	6	7	8	9	10	11	12	13	14
(1) Role breadth self efficacy	1													
(2) Absorption	0.37	1												
(3) Adaptability	0.09	0.38	1											
(4) Achievement orientation	0.12	0.24	0.35	1										
(5) Conflict management	0.30	0.47	0.48	0.47	1									
(6) Dedication	0.51	0.25	0.11	0.15	0.19	1								
(7) Empathy	0.62	0.60	0.37	0.23	0.52	0.34	1							
(8) Emotional self awareness	0.02	0.05	0.33	0.26	0.22	-0.09	0.02	1						
(9) Influence	0.22	0.33	0.44	0.38	0.52	0.19	0.30	0.23	1					
(10) Organizational awareness	0.05	0.23	0.38	0.37	0.26	0.13	0.16	0.34	0.29	1				
(11) Overall positive mood	0.18	0.39	0.30	0.46	0.49	0.23	0.36	0.11	0.38	0.39	1			
(12) Vigor	0.15	0.23	0.40	0.47	0.46	0.15	0.32	0.12	0.34	0.30	0.40	1		
(13) Compassion	0.17	0.10	-0.07	-0.01	0.02	0.24	0.25	-0.09	-0.02	-0.00	0.04	0.00	1	
(14) Vision	0.00	-0.02	0.11	0.10	0.23	-0.04	0.21	0.09	0.14	0.07	0.13	0.22	-0.07	1

### Common Method Bias (CMB)/Variance

Several steps were taken to mitigate, detect, and control for a common method bias (CMB). All survey items were carefully constructed and pre-tested, valid, multidimensional constructs were used (Huber, 1985). The scale anchors and format in the questionnaire were varied and a series of scale-validation processes were performed before distributions. The Harmon’s test results indicated that 28% of the variance is explained by a single factor. The correlations with the common variable, when including a marker variable, were 0.34, indicating a common method variance (CMV) of 11.6%, indicating that the study does not suffer from a CMB. Multicollinearity was examined through linear regression analysis on the study constructs and low variance inflation factors (VIFs) were found; nearly all VIFs were below three and only one construct; absorption (ABS) had indicators with VIFs above 5 (but less than 10). CMV was assessed using the Lindell and Whitney (2001) marker variable test. The results indicated that just over 7% of the common variance is between unrelated latent factors. To perform the marker test, a marker EI was added, with three questions (EMP6, OA6, INF6). The tests that were performed illustrate that although there is some bias in the model, the results are reliable and valid.

### Specification of the Structural Equation Model (SEM)

To test the hypotheses, a path model was specified in AMOS Version 18 and the maximum likelihood technique was used for its estimation. The structural equation model (SEM) tested the direct effects hypotheses of the emotional and social competencies and RBSE as related to the interpersonal environment, specifically the three sub-constructs: vision, compassion and overall positive mood and the direct effects of the perception of the interpersonal environment to engagement (absorption, dedication, and vigor).

## Results

**FIGURE 2:**
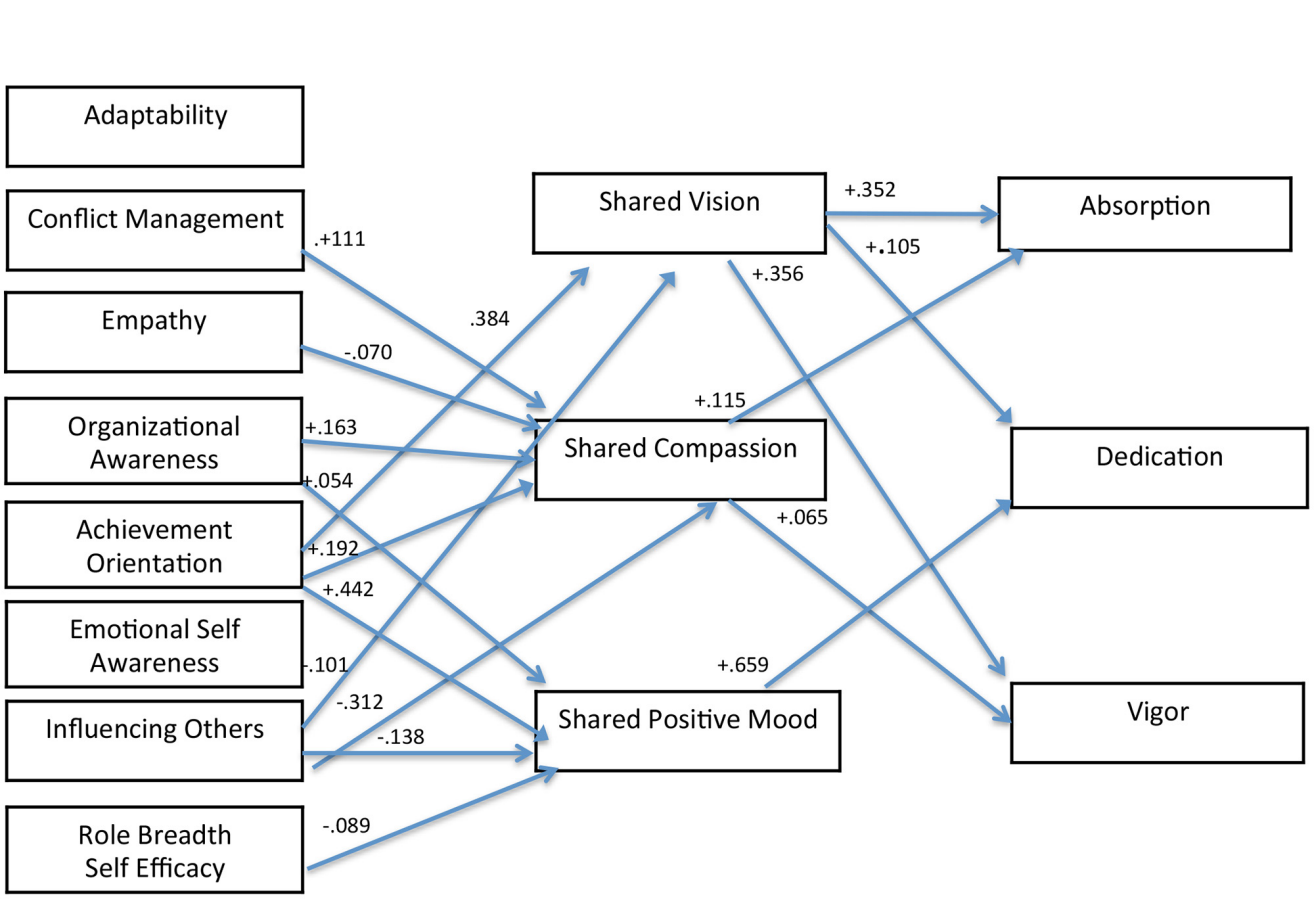
**Structural equation model (SEM) results**.

Of the 27 hypotheses, (17/27) were found to be statistically significant. Sixteen hypotheses were not supported and these were subsequently trimmed removing one non-significant path at a time from the fully identified model^2^. The final model is displayed in **Figure 2**. Hypothesis 1: Shared vision was positively related to all three sub-constructs of engagement: absorption (β = 0.352, *p* = 0.000) dedication (β = 0. 105, *p* = 0.005), and vigor (β = 0. 356, *p* = 0.000). Compassion was positively related to absorption (β = 0.115, *p* = 0.000) and vigor (β = 0.065, *p* = 0.009), while overall positive mood was positively related to dedication (β = 0.659, *p* = 0.000). Hypothesis 2: Only achievement orientation was positively related to shared vision (β = 0.384, *p* = 0.000), while influencing others was negatively related with shared vision (β = -0.101, *p* = 0.000). Hypothesis 3: The following competencies were positively related to compassion: achievement orientation (β = 0.192, *p* = 0.000), conflict management (β = 0.111, *p* = 0.003), and organizational awareness (β = 0.256, *p* = 0.000). Empathy (β = -0.070, *p* = 0.000) and influencing others was negatively related to compassion (β = -0.312, *p* = 0.000). Hypotheses 4: The following competencies were positively related to overall positive mood: organizational awareness (β = 0.054, *p* = 0.012), and achievement orientation (β = 0.442, *p* = 0.000). Influencing others was negatively related to overall positive mood (β = -0.138, *p* = 0.004). In summary, the relationship between achievement orientation and the perceived interpersonal environment was positive for all three sub-constructs and the relationship between influencing others and the perceived interpersonal environment was negative for all three sub-constructs. Hypothesis 5: RBSE was negatively related to overall positive mood (β = -0.089, *p* = 0.000).

## Discussion

The escalating organization investment in IT has not necessarily delivered the business value expected (Brynjolfsson, 1994), with less than a third of technology projects being delivered on budget or on time and almost a third canceled or never used (Johnson, 2009). Ellis (2009) claims that 75% of global companies admit to wasting one in three dollars spent on technology projects. There are many explanations for why IT has been aﬄicted with performance shortcomings. This study focuses on IT professionals and their lack of engagement. The goal of the research was to leverage the findings so that they could be applied to practice. This study is important because it examined if the perception of shared vision, compassion and overall positive mood would be positively related to engagement (absorption, dedication, and vigor) and explored how specific emotional and social competencies such as empathy, organizational awareness, emotional self-awareness, achievement orientation and conflict management might positively or negatively relate to the perception of shared vision, compassion, and overall positiveshort mood.

Christian et al. (2011) assert that specific job characteristics are strongly related to engagement. Rich et al. (2010) claim that the nature of employee’s behavioral contributions to their organizations is a function of job engagement. The focus on engagement relied on Schaufeli et al. (2002, p. 74) description of engagement as “a positive, fulfilling, work-related state of mind that is characterized by dedication, absorption, and vigor.” Engagement is, therefore not a momentary and specific state, but “a … persistent and pervasive affective-cognitive state not focused on any particular object, event, individual, or behavior.” The study findings reveal that perceptions of the interpersonal environment relate to how IT professionals engage at work. The study uncovered that the perception of shared vision related to all three of the components of engagement: dedication, vigor, and absorption. The study findings highlight that when a vision is created and assimilated within the organization people feel a part of a cause and are dedicated to the direction that leadership is driving. It is unfortunate, because in practice, leaders still fail to provide a clear vision to their people (Gallup Inc, 2013). Vision is seen as a way to “inspire others, to motivate action and to move with hope toward the future” (Farling et al., 1999, p. 53). Schaufeli and Bakker (2004) assert that absorption is characterized by being fully and enthusiastically engrossed in one’s work, whereby time flies by quickly. People feel excited when they have a shared vision and this vision connects the users. Next, the perception of shared compassion was related to vigor and absorption. These findings reveal that an interpersonal environment perceived as caring relates to the degree to which employees engage. The findings support Doty (2014) who claims that compassionate organizations have more engaged employees, generalizing that in a more compassionate workplace, employees are less stressed and more satisfied with their jobs, and turnover is lower. Building on this, Frost et al. (2000, p. 25) claim that compassion and care are not separate from being a professional or doing the work of the organization, but rather are a natural and living representation of people’s humanity in the workplace.” The study also uncovered that another sub-construct of the interpersonal environment; overall positive mood, is perceived by IT professionals to be positively related to dedication. An improvement in the attitude of the employees renders more produce positive outcomes, such as the employees’ increased performance and higher job satisfaction (Choi, 2012), leading to improved dedication. Additionally, employees who identify with the organization are likely to be more dedicated, especially if they think there is a future. An exchange relationship exists between the individual and the organization in which dedication is exchanged in the search for the results desired, such that employees become more engaged in their work (Flaherty and Pappas, 2000).

The research revealed that achievement orientation was the most significant emotional competency to positively relate to one’s perception of the interpersonal environment. Achievement orientation was perceived as positively related to all three of the sub-constructs of the interpersonal environment: shared vision, compassion, and overall positive mood. In the study hypothesis, it was originally hypothesized that achievement orientation would be perceived by IT professionals to be negatively related to compassion. The individualistic aspect of the work in IT informed this hypothesis. IT professionals enjoy working alone and feel like that can control their work elements, enhancing their sense of belonging and desire to work harder. However, the study findings revealed that the relationship of achievement orientation to be positively related to the IT professionals perception of compassion. This supports that even though IT professionals over achieve to promote themselves, they also do it for the good of the organization. Additionally, (McCullough, 2013) argues that employees with a high achievement orientation enjoy greater job satisfaction, which is related to a more positive perception of the interpersonal environment. High achievers tend to depend on certain factors under their own control and believe that success is a choice, while less achievement oriented individuals believe that success is outside of the individual’s control (McCullough, 2013).

Additionally, the study revealed that influencing others was perceived by IT professionals to be negatively related to all three of the sub-constructs of interpersonal environment: shared vision, compassion, and overall positive mood. This is supported by research on the personality of IT professionals. They tend to be introverted and work in a very specific discipline (similar to engineering and accounting). The creative, independent nature of the IT professional does not always align with traditional hierarchical, command-and-control organizations. The deployment of pure technical tasks typically does not span many boundaries beyond their organization. However, when IT professionals need to rely on their positional power to pressure others, this creates a dynamic that leads to disenfranchised employees (White, 2014). Influencing others does not appeal to most IT professionals. This finding may not be true for other working professionals and may be a distinctive finding to IT professionals.

Organization awareness was positively related to the IT professional’s perception of two sub-constructs of interpersonal environment: compassion and overall positive mood. Possessing higher levels of customer service is critical for IT professionals, given increasing demands from internal customers, such as marketing and sales departments, and pressure for more integration of IT with other organizational units (Lee et al., 1995). As observed by Ray et al. (2005), customer service has become a strategic imperative, linking a firm’s information technology resources and capabilities to achieving quality. IT professionals need to have keen insight into the strategies of multiple business units to be able to serve them effectively. The systems and software they create and implement serves the business and process needs of those within the organization. Having awareness of the organization builds trust and helps the IT professional to work smarter, not harder (Morton, 2014). McKenzie (2014) notes that working harder is a poor strategy and advises that a strategy that has become ingrained in the IT culture is to work smart and go home.

The research uncovered that conflict management is perceived by IT professionals to be positively related to compassion. Jordan and Troth (2004) claim that the act of integrating that occurs during conflict resolution is positive correlated with overall emotions. Since IT professionals are often misunderstood and negatively perceived by the business, IT professionals form a strong social identity with fellow workers to fight for their beliefs and demonstrate compassion for what they believe. In many organizations, IT is viewed as a cost center that does not provide much value. As a cost center, their funding is often reviewed and questioned and outsourcing of IT functions and/or downsizing are often perceived threats. To effectively deal with doubt and fear of their future employment, IT professionals must be ready and able to take on senior management as survival modus operandi.

There were three hypotheses that were not supported. Emotional self-awareness and overall positive mood were originally hypothesized to be negatively related, and in the study were not found to be related at all. The premise that IT professionals are forced to constantly reskill in the latest technologies to remain relevant was expected to be perceived as negatively related to overall positive mood. Since IT professionals are creative individuals, the idea of reskilling may appeal to some more than others, resulting in no significant relationship. With respect to empathy, a perceived negative relationship to compassion was revealed. Loundsbury et al. (2014) support this finding, claiming that IT professionals have personalities with significantly higher levels of agreeableness, tough-mindedness, and detachment. Further, they argue that IT professionals draw conclusions based on logic, facts, and data rather than feelings, values and intuition are more inclined to be analytical, objective and unsentimental when compared to other occupations.

Parker’s (1998) RBSE refers to employees’ perceived capability to carry out a broader and more proactive set of work tasks that extend beyond prescribed technical requirements. This study revealed that for IT professionals, no statistically significant relationship exists between RBSE and two of the three dimensions of the interpersonal environment (shared vision and compassion). The only finding, a negative relationship for RBSE and overall positive mood, was shown to be weak. The Institute for Management Excellence (2003) claims that 67% of IT professionals are introverted. The work in IT consists of independent tasks that require alone time, quiet concentration and attention focused on the work at hand, with little social interaction, which are best suited for introverts (Institute for Management Excellence, 2003). Given this, it is likely that their beliefs about their abilities to perform tasks beyond their prescribed roles may not be influenced by the perceived interpersonal environment. If the negative result for overall positive mood is real and not due to a methodological quirk, it could be that the exuberance of their coworkers actually makes the introverted IT professional somewhat uneasy, which makes them less willing to go beyond their prescribed roles. As such, reaching out and going beyond the task can be stressful for IT professionals, so they are likely to be low in RBSE. Since the RBSE of IT professionals are perceived to be negatively related to overall positive outlook, implementing interventions that focus on developing the personality characteristics associated with extraversion would support positivity in the workplace.

### Implications for Future Research and Practice

This study focused on what factors might positively relate to the engagement of IT professionals, one of the least engaged populations within organizations (Treadwell and Alexander, 2011). This work underscores the importance of emotional and social competencies and supports the research of McClelland (1973), Spencer and Spencer (1993), Goleman (1998), and Boyatzis (2008) with respect to the impact of competencies on the interpersonal environment specifically, and more generally, the organizational environment as a whole. The research study was motivated by the importance of understanding how emotional and social competencies might affect the interpersonal environment, which in turn, might affect employee engagement. The negative cost to having employees that are not engaged includes lower productivity and higher turnover (Bassi and McMurrer, 2007). By contributing knowledge, practice could be positively impacted, further supported by Ramirez et al. (2001) who claim that IT organizations that earn higher returns are ones who implement employee engagement initiatives. This study provides an increased understanding of the behaviors that relate to the IT professional’s perception of the interpersonal environment, and in turn, how the interpersonal environment might impact the engagement of IT professionals. This knowledge can be leveraged in practice to increase the percentage of IT professionals engaged at work. Using the specific behaviors identified as positively impacting the interpersonal environment, practitioners can apply this knowledge to several of their Human Capital Management processes. In the hiring process, organizations should implement an interview protocol that supports identifying candidates that are achievement oriented. Another behavior, organizational awareness, was significantly related to two interpersonal environment constructs: compassion and overall positive mood. These two behaviors should be added to talent development criteria and promotional processes. Having individuals with these behaviors will positively relate to the perception of the organizational environment, which in turn, will positively relate to employee engagement. The study findings also support Stajkovic and Luthans (1998) and Boyatzis (2008) who claim that the perception of the interpersonal environment impacts the level of employee engagement. On an organizational level, the study findings have important implications for those involved in the strategic planning of human capital. Leaders should be encouraged to leverage the findings to improve management practices that influence the interpersonal environment.

The research uncovered that engagement is positively impacted by each of the distinctive components of the interpersonal environment (vision, compassion, and overall positive mood). As a result, it is recommended that leaders actively communicate the organization’s vision and ensure that employees understand and align with such vision. The study findings highlight that when a vision is created and assimilated within the organization, people feel a part of a cause and are dedicated to the direction that leadership is driving. Yet, leaders still fail to provide a clear vision to their people (Gallup Inc, 2013). Vision is seen as a way to “inspire others, to motivate action, and to move with hope toward the future” (Farling et al., 1999, p. 53). People feel excited when they have a shared vision. Leaders should be encouraged to evaluate the organization’s culture to gain an understanding if compassion is valued, supported, and nurtured. This study revealed that compassion is significantly related to absorption and vigor, demonstrating that having employees passionate about their organization impacts how much they engage. The findings support Doty (2014) who claims that compassionate organizations have more engaged employees, generalizing that in a more compassionate workplace, employees are less stressed and more satisfied with their jobs, and turnover is lower. Finally, the findings revealed that an employee’s overall positive mood was significantly related to their level of dedication. Dedicated employees become more absorbed in their work, resulting in greater work outcomes. Dedicated employees envision a future with the organization. Therefore, it is recommended that organizations create career paths and provide internal mentors to support employees to develop personal career plans. Researchers are encouraged to further explore how specific emotional and social competencies and RBSE affect engagement. Future inquiry could focus on a broader set of factors within the interpersonal environment to increase understanding of the IT professional. Finally, the research could be expanded to include other types of workers, outside of IT.

### Limitations

This study has some limitations that should be noted. Although the focus on IT professionals was deliberate, it may not be generalizable to all IT professionals because the respondents were recruited and the sample was drawn from a few global companies and IT professional associations in the United States and Canada. Additional data providing further insight into the respondents such as industry type, geography, etc. could have been collected. A more global sample may have produced different results. Further, the data is self-reported and a 360° or manager feedback mechanism was not used, which could have provided a broader perspective. The survey instrument consisted of four widely used measures that were carefully selected and integrated to best reflect the theories that informed this research. Three of the measurement constructs were used in their entirety. Due to the size of the ESCI-U construct, only seven of the 14 competencies were chosen. By not using the entire measurement instrument, the overlooked dimensions may have proven significant to the findings. With respect to the findings on RBSE, the results inform that there is a RBSE has a relationship with shared vision, compassion, and positive mood.

## Conclusion

This study on employee engagement is unique in its focus on IT professionals – in particular with respect to examining which emotional and social competencies and RBSE relate to aspects of the interpersonal environment and what aspects of the interpersonal environment might relate to engagement. This research can help practitioners consider which factors might help improve engagement, increase innovation, and improve performance.

## Conflict of Interest Statement

The author declares that the research was conducted in the absence of any commercial or financial relationships that could be construed as a potential conflict of interest.

## Footnotes

1 It should be noted I held the tolerance for the factors to 0.7, but the ESCI instrument has been determined valid at a 0.4 or higher level. Taking this into consideration, I performed ran a second EFA leaving in the lower factor-loading as acceptable, I found that fewer questions were eliminated from the constructs, but my findings did not significantly change.

2 The non-significant paths were: (1) adaptability positively relates to vision, compassion and overall positive mood, (2) conflict management negatively relates to vision, (3) empathy positively relates to vision and compassion, (4) organizational awareness positively relates to vision, (5) emotional self-awareness positively relates to vision, (6) emotional self-awareness positively relates to compassion, (7) emotional self-awareness negatively relates to overall positive mood. (8) RBSE positively relates to vision and compassion, (9) compassion relates to dedication, (10) overall positive mood will positively relates to vigor, and (11) overall positive mood will positively relates to absorption.

## References

[B1] Agarwal R. Ferratt T. W. (2002). Enduring practices for managing IT professionals. *Commun. ACM* 45 73–79. 10.1145/567498.567502

[B2] Alvesson M. Robertson M. Swan J. (2001). “The best and the brightest: the role of elite identity in knowledge-intensive companies,” in *Proceedings of the Critical Management Studies Conference* (Manchester: Manchester School of Management).

[B3] Ang S. Slaughter S. A. (2000). “The missing context of information technology personnel: a review and future directions for research,” in *Framing the Domain of IT Management: Projecting the Future through the Past*, ed. Zmud R. W. (Cincinnati, OH: Pinnaflex Education Resources, Inc).

[B4] Aronoff J. Stollak G. Woike B. (1994). Affect regulation and the breadth of interpersonal engagement. *J. Pers. Soc. Psychol.* 67 105–114. 10.1037/0022-3514.67.1.105

[B5] Bassi L. McMurrer D. (2007). *Maximizing Your Return on People.* Harvard Business Review, Reprint R0703H Cambridge, MA: Harvard Business Press. 17348175

[B6] Bates S. (2004). Getting engaged. *HR Magazine* 49 44–51.

[B7] Baumeister R. Leary M. (1995). The need to belong: desire for interpersonal attachments as a fundamental human motivation. *Psychol. Bull.* 117 497–529. 10.1037/0033-2909.117.3.497 7777651

[B8] Becker B. Huselid M. (2006). Strategic human resources management: where do we go from here? *J. Manag.* 32 898–925. 10.1177/0149206306293668

[B9] Bentler P. M. (1990). Comparative fit indexes in structural models. *Psychol. Bull.* 107 238–246. 10.1037/0033-2909.107.2.238 2320703

[B10] Betancourt H. (1990). An attribution-empathy model of helping behavior emotional and social intentions and judgments of help giving. *Pers. Soc. Psychol. Bull.* 16 573–591. 10.1177/0146167290163015

[B11] Boyatzis R. E. (1982). *The Competent Manager: A Model for Effective Performance.* New York, NY: John Wiley & Sons.

[B12] Boyatzis R. E. (2001). “How and why individuals are able to develop emotional intelligence,” in *The Emotionally Intelligent Workplace: How to Select for, Measure, and Improve Emotional Intelligence in Individuals, Groups, and Organizations*, eds Cherniss C. Goleman D. (San Francisco, CA: Jossey-Bass), 234–253.

[B13] Boyatzis R. E. (2006). Intentional change theory from a complexity perspective. *J. Manag. Dev.* 25 607–623. 10.1108/02621710610678445

[B14] Boyatzis R. E. (2008). Competencies in the twenty-first century. *J. Manag. Dev.* 27 5–12. 10.1108/02621710810840730

[B15] Boyatzis R. E. (2009). Competencies as a behavioral approach to emotional intelligence. *J. Manag. Dev.* 28 749–770. 10.1108/02621710910987647

[B16] Boyatzis R. E. (2013). “When pulling to the negative emotional attractor is too much or not enough to inspire and sustain outstanding leadership,” in *The Fulfilling Workplace: The Organization’s Role in Achieving Individual and Organizational Health*, eds Burke R. Cooper C. Woods G. (London: Gower Publishing), 139–150.

[B17] Boyatzis R. E. Goleman D. (1996). *Emotional Competency Inventory*. Boston, MA: The Hay Group.

[B18] Boyatzis R. E. Goleman D. Hay Acquisition (2001/2007). *Emotional and Social Competency Inventory.* Boston, MA: The Hay Group.

[B19] Boyatzis R. E. Massa R. Good D. (2012). Emotional, social and cognitive intelligence as predictors of sales leadership performance. *J. Leadersh. Organ. Stud.* 19 191–201. 10.1177/1548051811435793

[B20] Boyatzis R. E. Smith M. L. Beveridge A. (2013). Coaching with compassion: inspiring health, well-being and development in organizations. *J. Appl. Emot. Behav. Sci.* 49 153–178. 10.1177/0021886312462236

[B21] Browne M. W. Cudeck R. (1993). “Alternative ways of assessing model fit,” in *Testing Structural Equation Models*, eds Bollen K. A. Long J. S. (Beverly Hills, CA: Sage), 136–162.

[B22] Brynjolfsson E. (1994). Information assets, technology, and organization. *Manag. Sci.* 40 1645–1662. 10.1287/mnsc.40.12.1645

[B23] Brynjolfsson E. Hitt L. (2000). Beyond computation: information technology, organizational transformation and business performance. *J. Econ. Perspect.* 14 23–48. 10.1257/jep.14.4.23

[B24] Capretz L. F. (2002). Personality types in software engineering. *Int. J. Hum. Comput. Stud.* 58 207–214. 10.1016/S1071-5819(02)00137-4

[B25] Choi S. (2012). Demographic diversity of managers and employee job satisfaction: empirical analysis of the federal case. *Rev. Public Pers. Adm.* 33 275–298. 10.1177/0734371X12453054

[B26] Christian M. Garza A. Slaughter J. (2011). Work engagement: a quantitative review and test of its relations with task and contextual performance. *Pers. Psychol.* 64 89–136. 10.1111/j.1744-6570.2010.01203.x

[B27] Corporate Leadership Council. (2004). *Driving Performance and Retention through Employee Engagement.* Washington, DC: Corporate Executive Board.

[B28] Dietze E. Orb A. (2000). Compassionate care: a moral dimension of nursing*. *Nurs. Inq.* 7 166–174. 10.1046/j.1440-1800.2000.00065.x

[B29] Den Hartog D. N. Belschak F. D. (2007). Personal initiative, commitment and affect at work. *J. Occup. Organ. Psychol.* 80 601–622. 10.1348/096317906X171442

[B30] Doty J. (2014). Mindfulness and compassion are good for business bottom line. *Australian Broadcasting System.* (accessed September 7 2014).

[B31] Dutton J. E. Frost P. J. Worline M. C. Lilius J. M. Kanov J. M. (2002). Leading in times of trauma. *Harv. Bus. Rev.* 80 54–61. 12964467

[B32] Eisenberger R. Huntington R. Hutchison S. Sowa D. (1986). Perceived organizational support. *J. Appl. Psychol.* 71 500–507. 10.1037/0021-9010.71.3.500

[B33] Elliot A. J. (2005). “A conceptual history of the achievement goal construct,” in *Handbook of Competence and Motivation*, eds Elliot A. J. Dweck C. S. (New York, NY: Guilford Press), 52–72.

[B34] Ellis K. (2009). *Business Analysis Benchmark 2009: The Pass to Success*. New Castle, DE: IAG Consulting.

[B35] Ely R. (1995). The power of demography: women’s social constructions of gender identity at work. *Acad. Manag. J.* 38 589–634. 10.2307/256740

[B36] Eve-Cahoon H. (2003). Editorial: understanding the introvert preference. *J. Nurs. Educ.* 42 191–193. 12769421 10.3928/0148-4834-20030501-03

[B37] Farling M. Stone A. Winston B. (1999). Servant leadership: setting the stage for empirical research. *J. Leadersh. Stud.* 6 49–72. 10.1177/107179199900600104

[B38] Flaherty K. E. Pappas J. M. (2000). The role of trust in salesperson-sales manager relationships. *J. Pers. Selling Sales Manag.* 20 271–278.

[B39] Floyd F. J. Widaman K. F. (1995). Factor analysis in the development and refinement of clinical assessment instruments. *Psychol. Assess.* 7 286–299. 10.1037/1040-3590.7.3.286

[B40] Fornell C. Larcker D. (1981). Evaluating structural equation models with unobservable variables and measurement error. *J. Mark. Res.* 18 39–50. 10.2307/3151312

[B41] Fredrickson B. Losada M. (2005). Positive affect and the complex of human flourishing. *Am. Psychol.* 60 678–686. 10.1037/0003-066X.60.7.678 16221001 PMC3126111

[B42] Frost P. Dutton J. Worline M. Wilson A. (2000). “Narratives of compassion in organizations,” in *Emotions in Organizations*, ed. Fineman S. (London: Sage), 25–45.

[B43] Gagnon D. Moore G. Shanmuganathan G. (2014). Factors mediating between employee strategy awareness and commitment to organizational success. *J. Manag. Sustain.* 4 24–31. 10.5539/jms.v4n4p24

[B44] Gallup Inc. (2013). *State of the American Workplace.* Report. Available at: http://www.gallup.com/poll/165269/worldwide-employees-engaged-work.aspx?version=print

[B45] Gefen D. Straub D. Boudreau M. (2000). Structural equation modeling and regression: guidelines for research practice. *Commun. Assoc. Inf.Syst.* 4:7.

[B46] George J. (1991). Effects of positive mood on prosocial behaviors at work. *J. Appl. Psychol.* 76 299–307. 10.1037/0021-9010.76.2.299

[B47] Goleman D. (1998). *Working with Emotional Intelligence.* New York, NY: Bantam.

[B48] Goleman D. Boyatzis R. McKee A. (2013). *Primal Leadership, With a New Preface by the Authors: Unleashing the Power of Emotional Intelligence.* Boston, MA: Harvard Business Press.

[B49] Gross J. J. (1998). Antecedent- and response-focused emotion regulation: divergent consequences for experience, expression, and physiology. *J. Pers. Soc. Psychol.* 74 224–237. 10.1037/0022-3514.74.1.224 9457784

[B50] Haidt J. (2012). *The Righteous Mind.* New York, NY: Pantehon Books.

[B51] Hair J. F. Black W. C. Babin B. J. Anderson R. E. (2010). *Multivariate Data Analysis*, 7th Edn Upper Saddle River, NJ: Prentice Hall.

[B52] Hitt L. Brynjolfsson E. (1994). “The three faces of IT value: theory and evidence,” in *Proceedings of the 15th International Conference on Information Systems*, Vancouver, BC, 263–277.

[B53] Hu L. Bentler P. (1998). Fit indices in covariance structure modeling: sensitivity to underparameterized model misspecification. *Psychol. Methods* 3 424–453. 10.1037/1082-989X.3.4.424

[B54] Huber P. (1985). Projection pursuit. *Ann. Stat.* 13 435–475. 10.1214/aos/1176349519

[B55] Iacono S. Weisband S. (1997). “Developing trust in virtual teams,” in *Proceedings of the 30th Annual Hawaii International Conference on System Sciences (HICSS)*, Maui, HI, 412–420. 10.1109/HICSS.1997.665615

[B56] Institute for Management Excellence (2003). *Differences Between “Computer” Folks and the General Population.* Available at: http://www.itstime.com/jul2003.htm (accessed April 14 2006).

[B57] Jarvenpaa S. L. Knoll K. Leidner D. E. (1998). Is anybody out there? Antecedents of trust in global virtual teams. *J. Manag. Inf. Syst.* 14 29–64.

[B58] Johnson G. (2004). Otherwise engaged. *Training* 41 4–17.

[B59] Johnson J. (2009). *Chaos Summary.* The Standish Group Report. Available at: http://www.standishgroup.com/sample_research

[B60] Jordan P. J. Troth A. C. (2004). Managing emotions during team problem solving: emotional intelligence and conflict resolution. *Hum. Perform.* 17 195–218. 10.1207/s15327043hup1702_4

[B61] Judge T. (2002). Relationship of personality to performance motivation: a meta-analytic review. *J. Appl. Psychol.* 87 797–807. 10.1037/0021-9010.87.4.797 12184582

[B62] Kahnweiler J. (2013). The power of introverts. *Administrative Professional Today* August 2013: 1+; Business Insights, Essentials: 14 April 2015.

[B63] Kanov J. Maitlis S. Worline M. Dutton J. (2004). Compassion in organizational life. *Am. Emot. Soc. Sci.* 47 808–827. 10.1177/0002764203260211

[B64] Kowalski B. (2003). The engagement gap. *Training* 40 62.

[B65] Lee D. M. Trauth E. M. Farwell D. (1995). Critical skills and knowledge requirements of IS professionals: a joint academic/industry investigation. *MIS Q.* 19 313–340. 10.2307/249598

[B66] Lilius J. Worline M. Dutton J. Kanov J. Frost P. Maitlis S. (2003). What good is compassion at work? *Ann. Arbor.* 1001 48109–41109.

[B67] Lindell M. Whitney D. (2001). Accounting for common method variance in cross- sectional research designs. *J. Appl. Psychol.* 86 114–121. 10.1037/0021-9010.86.1.114 11302223

[B68] Loundsbury J. Foster N. Patel H. Carmody P. Gibson L. Stairs D. (2012). An investigation of the personality traits of scientists versus non-scientists and their relationship with career satisfaction. *R&D Manag.* 42 47–59. 10.1111/j.1467-9310.2011.00665.x

[B69] Loundsbury J. Sundstrom E. Levy J. J. Gibson L. W. (2014). Distinctive personality traits of information technology professionals. *Comput. Inf. Sci.* 7 38–48. 10.5539/cis.v7n3p38

[B70] Mahon E. Taylor S. Boyatzis R. (2014). Antecedents of organizational engagement: exploring vision, mood, and perceived support with emotional intelligence as a moderator. *Front. Psychol.* 5:1322 10.3389/fpsyg.2014.01322 PMC4235263 25477845

[B71] McClelland D. C. (1961). *The Achieving Society.* New York, NY: D. Van Nostrand 10.1037/14359-000

[B72] McClelland D. (1973). Testing for competence rather than intelligence. *Am. Psychol.* 28 1–14. 10.1037/h0034092 4684069

[B73] McCullough M. (2013). “Achievement orientation,” in *Proceedings of the Academy of Organizational Culture, Communications and Conflict of Allied Academies International Conference*, Vol. 18 Reno, NV, 23–27.

[B74] McKenzie P. (2014). *Work Less, Get More Done: Analytics for Maximizing Productivity.* Available at: http://www.kalzumeus.com/2009/10/04/work-smarter-not-harder/ (accesses April 22 2014).

[B75] Morton K. (2014). *Work Smart, Not Hard: Be More Productive by Spending Less Time in Front of Your Computer.* Available at: http://theprogrammersparadox.blogspot.com/search?updated-min=2012-01-01T00:00:00-05:00&updated-max=2013-01-01T00:00:00-05:00&max-results=26 (accessed April 7 2014).

[B76] Nunnally J. (1978). “Fundamentals of factor analysis,” in *Psychometric Theory*, 2nd Edn, ed. Nunnally J. (New York, NY: McGraw-Hill Book Company),327–404.

[B77] Ostroff C. Kinicki A. J. Tamkins M. M. (2003). “Organizational culture and climate,” in *Handbook of Psychology: Industrial and Organizational Psychology*, eds Borman W. C. Ilgen D. R. Klimoski R. J. (Hoboken, NJ: Wiley), 565–593.

[B78] Parker S. (1998). Enhancing role breadth self-efficacy: the roles of job enrichment and other organizational interventions. *J. Appl. Psychol.* 83 835–852. 10.1037/0021-9010.83.6.835 9885197

[B79] Pavlovich K. Krahnke K. (2012). Empathy, connectedness and organisation. *J. Bus. Ethics* 105 131–137. 10.1007/s10551-011-0961-3

[B80] Pratt M. G. Rockmann K. W. Kaufmann J. B. (2006). Constructing professional identity: the role of work and identity learning cycles in the customization of identity among medical residents. *Acad. Manag. J.* 49 235–262. 10.5465/AMJ.2006.20786060

[B81] Preenan P. Van Vianen A. De Pater I. (2014). Challenging assignments and activating mood: the influence of goal orientation. *J. Appl. Soc. Psychol.* 44 650–659. 10.1111/jasp.12256

[B82] Quinn J. F. (2014). The affect of vision and compassion upon role factors in physician leadership. *Front. Psychol.* 6:442 10.3389/fpsyg.2015.00442 PMC4424809 26005425

[B83] Rahim M. A. Garrett J. E. Buntzman G. F. (1992). Ethics of managing interpersonal conflict in organizations. *J. Bus. Ethics* 11 423–432. 10.1007/BF00870554

[B84] Ramirez R. Kraemer K. L. Lawler E. (2001). *The Contribution of Information Technology Investments to Firm Performance: Influence of Management Practices.* Working paper. Center for Research on Information Technology and Organizations, University of California, Irvine, CA.

[B85] Ray G. Muhanna W. Barney J. (2005). Information technology and the performance of the customer service process: a resource-based analysis. *MIS Q.* 29 625–652.

[B86] Regan D. T. Totten J. (1975). Empathy and attribution: turning observers into actors. *J. Pers. Soc. Psychol.* 32 850–856. 10.1037/0022-3514.32.5.850 1185516

[B87] Rich B. Lepine J. Crawford E. (2010). Job engagement: antecedents and effects on job performance. *Acad. Manag. J.* 53 617–635. 10.5465/AMJ.2010.51468988

[B88] Saks A. (2006). Antecedents and consequences of employee engagement. *J. Manag. Psychol.* 21 600–619. 10.1108/02683940610690169

[B89] Schaufeli W. B. Bakker A. (2004). Job demands, job resources, and their relationship with burnout and engagement: a multi-sample study. *J. Organ. Behav.* 25 293–315. 10.1002/job.248

[B90] Schaufeli W. B. Salanova M. Gonzalez-Roma V. Bakker A. B. (2002). The measurement of engagement and burnout: a two sample confirmatory analytic approach. *J. Happiness Stud.* 3 71–92. 10.1023/A:1015630930326

[B91] Seppala E. Rossomando T. Doty J. (2013). Social connection and compassion: important predictors of health and well-being. *Soc. Res.* 80 411.

[B92] Spencer L. M. Jr. Spencer S. M. (1993). *Competence at Work: Models for Superior Performance.* New York, NY: Wiley.

[B93] Stajkovic A. Luthans F. (1998). Social cognitive theory and self-efficacy: going beyond traditional motivational and emotional and social approaches. *Organ. Dyn.* 26 62–73. 10.1016/S0090-2616(98)90006-7

[B94] Steiger J. H. Lind J. C. (1980). “Statistically-based tests for the number of common factors,” in *Paper Presented at the Annual Spring Meeting of the Psychometric Society*, Iowa City, IA.

[B95] Straub D. Boudreau M. Gefen D. (2004). Validation guidelines for IS positivist research. *Commun. Assoc. Inf. Syst.* 13 380–427.

[B96] Tajfel H. (1978). *Social Identity and Intergroup Relations.* Cambridge: Cambridge University Press.

[B97] Towers Watson (2012). *Engagement at Risk: Driving Strong Performance in a Volatile Global Environment.* Global Workforce Study Report. New York.

[B98] Treadwell D. Alexander P. (2011). *Money isn’t all that Matters: Strategies for Attracting and Retaining Technical Professionals.* Skillman, NJ: Blessing White.

[B99] Tucker L. Lewis C. (1973). A reliability coefficient for maximum likelihood factor analysis. *Psychometrika* 38 1–10. 10.1007/BF02291170

[B100] Webster J. Martocchio J. (1992). Microcomputer playfulness: development of a measure with workplace implications. *MIS Q.* 16 201–226. 10.2307/249576

[B101] Weick K. E. (1995). *Sensemaking in Organizations.* Thousand Oaks, CA: Sage Publications.

[B102] White B. (2014). The art of influencing without authority. *Blessing White Research Report.* (accessed June 2014).

[B103] Woodruff C. (1980). Data processing people — are they really different? *Inf. Manag.* 3 133–139. 10.1016/0378-7206(80)90019-1

[B104] Wynekoop J. L. Walz D. B. (1998). Revisiting the perennial question: are IS people different? *Database Adv. Inf. Syst.* 29 62–72. 10.1145/298752.298759

